# The Effect of Age on the Regenerative Potential of Adipose Stem-cell-derived Apoptotic Extracellular Vesicles in Rat Skin Wound Healing

**DOI:** 10.7150/ijms.94755

**Published:** 2024-05-28

**Authors:** Kaixin Yan, Lu Han, Siwei Xu, Linli Jiang, Xinnan Chang, Hui Li, Lei Liu

**Affiliations:** 1 State Key Laboratory of Oral Disease & National Center for Stomatology & National Clinical Research Center for Oral Diseases, West China School of Stomatology, Sichuan University, Chengdu 610041, Sichuan, China.; 2 Department of Oral and Maxillofacial Surgery, West China Hospital of Stomatology, Sichuan University, Chengdu 610041, Sichuan, China.; 3 Department of Periodontics and Oral Medicine, University of Michigan School of Dentistry, Ann Arbor, MI, United States.; 4 Department of Biomaterials Sciences and Prosthodontics, School of Dentistry, University of Michigan, Ann Arbor, 48109, MI, United States.

**Keywords:** aging, apoptotic extracellular vesicles, adipose stem cell, endothelial cells, fibroblasts, skin wound healing

## Abstract

**Introduction:** Skin, being the body's largest organ, is susceptible to injuries. Despite the adoption of common treatments such as debridement, wound dressing, and infection control measures for skin injuries, the outcomes remain unsatisfactory, especially in diabetic patients or elderly patients. The use of adipose stem cell-derived apoptotic extracellular vesicles (apoEVs-ASCs) has been shown great therapeutic potential in wound repair. The effect of the donor age on the biological properties and functions of apoEVs-ASCs has not been reported.

**Methods:** In this study, we isolated apoEVs-ASCs from young and aged rats. Transmission electron microscopy (TEM) and nanoparticle tracking analysis (NTA) were applied for the characteristics of apoEVs-ASCs. For aged and young apoEVs-ASCs groups, the proliferative and migration abilities *in vitro*, and wound healing function *in vivo* were contrastively evaluated and quantified for statistical analysis.

**Results:** Our results showed that both young and aged apoEVs-ASCs induced skin healing and reduced scar formation. In addition, young apoEVs-ASCs had significantly higher proliferation, migration of fibroblasts and endothelial cells, and increased neo-angiogenesis ability, when compared with that of aged apoEVs-ASCs.

**Conclusion:** Young apoEVs-ASCs should be employed for wound repair, which is associated with its superior promoting effect on wound healing.

## 1. Introduction

As the largest human body organ, the skin plays a vital role in maintaining homeostasis and protecting the body from the external environment 1, 2. Skin injuries can be caused by many destructive external stimuli, such as burns, trauma, disease, and surgery. Wound healing is a complex process, which involves homeostasis, inflammation, proliferation, and remodeling.

Despite the adoption of common treatments such as cleaning, debridement, wound dressing, and infection control measures for skin injuries, the outcomes remain unsatisfactory, particularly in patients with specific conditions, such as vascular diseases, elderly age, diabetes mellitus, severe malnutrition, and radiation therapy. Failure to effectively treat skin wounds can lead to unhealing wounds, infection, function loss, and even amputation. Even when the wound is healed, hypertrophic and atrophic scar formation remains a complex issue, which could lead to cosmetic concerns, functional difficulties, and even psychological impacts. Impaired wound healing and its medical complications pose a significant burden on patients, medical institutions, and society 3-7. Therefore, the development of more effective treatments for addressing the complexities of wound healing remains challenging.

In recent years, stem cell-based therapy has been shown to play a significant role in tissue restoration and bone regeneration, to increase wound healing or restore bone 8-12. Mesenchymal stem cells (MSCs), embryonic stem cells (ESCs), and induced pluripotent stem cells (iPSCs) have been commonly used 13-14. However, issues associated with cell transplantation therapy, such as immunological incompatibility, cell death, abnormal cell differentiation and proliferation, viral transmission from animals to humans, and ethical problems 15. Owing to the aforementioned limitations of cell transplantation therapy, an alternative strategy for wound healing is urgently needed.

Recent studies have demonstrated a paracrine mechanism of MSCs for its therapeutic efficacy via extracellular vehicles (EVs). Interestingly, stem cells have been shown to undergo apoptosis in a short time and then paracrine extensive apoptotic extracellular vesicles (apoEVs) containing various cellular components including proteins, nucleic acids, and lipid profiles, after *in vivo* application 16-20. ApoEVs are key regulators of intercellular communications between stem cells and target cells and can participate in multiple physiological and pathological processes, such as inflammation 16, immune responses 22, 23, coagulation 24, tumorigenesis 25-27 and tissue regeneration 28-30. ApoEVs can be classified into apoptotic bodies (1-5um), apoptotic microvesicles (0.1-1um), and apoptotic exosome-like vesicles (< 150nm) 31.

The use of apoEVs as therapeutic agents has been an area of interest in skin regeneration. For example, apoEVs derived from adipose tissue have been shown to promote skin wound healing 32. Recent studies have reported that apoEVs derived from umbilical cord mesenchymal stem cells ameliorate cutaneous wound healing in type 2 diabetic mice via macrophage pyroptosis inhibition 33. Furthermore, a study demonstrated that apoptotic bodies derived from mesenchymal stem cells promote wound healing and hair growth through the Wnt/β-catenin pathway 34. These benefits of apoEVs have shown clinical applications for treating problematic wound healing.

In contrast with other stem cells, adipose stem cells (ASCs) have many more advantages, such as their prompt availability, abundant adipose tissue, and ease of tissue harvesting 35. Moreover, ASCs can secrete multiple growth factors, cytokines, and chemokines, contributing to skin repair and regeneration. ApoEVs-ASCs have attracted growing attention in the field of skin wound healing and scar treatment.

Although these achievements have gained vast attention, there are still barriers that restrict their clinical application. The therapeutic effects have been unsatisfactory due to the quantity and quality of the stem cells, which may be affected by the age of the donor. Stem cells undergo significant changes with aging. All these alterations contributed to low stem cell numbers and deteriorated function 36. Does donor age affect the *in vitro* and *in vivo* regenerative potential of apoEVs-ASCs in an animal model of skin injury? Till now, the effect of apoEVs-ASCs derived from young and aged rats on skin wound healing remains unknown.

In this study, we aimed to compare the protective effects between apoEVs-ASCs derived from young and aged rats in wound injury and elucidate the potential mechanisms. ApoEVs-ASCs were isolated from healthy rats of 3 weeks old and 22 months old, respectively, and administered to young rats with skin injuries.

## 2. Materials and methods

### 2.1 Animals

Sprague Dawley (SD) rats were purchased from Chengdu Dashuo Experimental Animal Co., Ltd (China). Young (3 weeks old), aged (22 months old), and 8 weeks old SD male rats were used for all experiments. All rats were housed in pathogen-free conditions, maintained on a standard 12-hour light-dark cycle, and received food and water at libitum. This study was performed in strict accordance with the recommendations in the Guide for the Care and Use of Laboratory Animals at Sichuan University. Animal procedures were performed according to protocols approved by the Animal Ethics Committee at Sichuan University (WCHSIRB-D-2022-617). All animal experiments followed the ARRIVE (Animal Research: Reporting of *In Vivo* Experiments) guidelines.

### 2.2 ASCs harvesting from rat inguinal adipose tissue

Inguinal adipose tissue was isolated from 3-week-old and 22-month-old male SD rats. The adipose tissue was rinsed three times with sterile cold phosphate-buffered saline (PBS), then cut into small pieces (1mm^3^), and digested in 0.2 % type-I collagenase (Sigma, MO, USA) for 1 h at 37°C, before centrifugation at 1000 rpm for 10 min. ASCs were cultured in minimum essential medium α (α-MEM, Thermo Scientific HyClone, UT, USA) with 15% fetal bovine serum (FBS, Lonsera, Uruguay, South America) and 1% penicillin-streptomycin liquid (Beyotime, Shanghai, China) and incubated at 37°C and 5% CO_2_. The culture medium was changed for the first time after 48 hours and then was changed every two days. Cells between passages 2 and 3 from aged and young rats, grown as a monolayer to 80% confluence, were used in subsequent examinations. The senescence proportion of ASCs was detected by SA-β-gal assay.

### 2.3 Isolation and characterization of apoEVs-ASCs

ApoEVs-ASCs were isolated by using Qu's protocol 37. Firstly, ASCs from young and aged rats were treated with STS (Cell Signaling Technology, USA) at 0.5 μM for 16-18 h to induce apoptosis. The apoptotic rate of ASCs was detected by TUNEL staining. When the apoptotic rate of ASCs reached 90%, the supernatant was collected and centrifuged at 800g for 10 min as well as 2000g for 10 min at 4°C to remove cells and debris. Then, the supernatant was further centrifuged at 16,000g for 30 min at 4°C, and the pellet was washed twice in PBS. The isolated apoEVs-ASCs from young and aged rats were suspended in 100 μl PBS and stored at -80°C for further study.

The protein concentration of apoEVs-ASCs was measured by the BCA protein assay kit (KeyGEN BioTECH, China) using the manufacturer's protocol. For morphology observation, young and aged apoEVs-ASCs were loaded onto formvar carbon-coated grids, negatively stained with aqueous phosphotungstic acid at room temperature for 60 s, and imaged by transmission electron microscope (TEM, Tecnai G2 F20 S-Twin, USA). The size distribution was measured by nanoparticle tracking analysis (NTA) by using Zeta View (Particle Metrix, Germany).

### 2.4 Cell culture

Fibroblasts (HFF cell line) and endothelial cells (HUVEC cell line) were purchased from the Cell Bank of the Chinese Academy of Sciences (China). HFFs and HUVECs were seeded in α-MEM and DMEM, supplemented with 10% FBS, 1% penicillin-streptomycin liquid, and cultured in a 5% CO_2_-95% air atmospheric condition at 37°C. When cells reached 85-95% confluence, they were trypsinized and passaged at a 1:2 ratio.

### 2.5 CCK-8 assay

Fibroblasts and endothelial cells were seeded at 1×10^3^ cells per well into 96-well plates. Cells were divided into the PBS group (100 μL PBS per well), the aged apoEVs-ASCs group (100 μL PBS containing 2.5 μg aged apoEVs-ASCs per well), and the young apoEVs-ASCs group (100 μL PBS containing 2.5 μg young apoEVs-ASCs per well). The culture medium per well was changed every 2 days to maintain the treatment effect. The optical density (OD) value was detected at 450 nm using the Multiskan Go Spectrophotometer (Thermo Fisher Scientific) from day 1 to day 5.

### 2.6 Cell scratch assay

To investigate the migration ability of fibroblasts and endothelial cells, fibroblasts and endothelial cells were seeded at 1×10^5^ cells per well into 24-well plates. When cells contacted and formed a confluent monolayer, pipette tips were used to scratch according to the marked line. Images at this time (0 h) were captured by an inverted microscope (Olympus, Japan). Then, cells were divided into the PBS group (500 μL PBS per well), the aged apoEVs-ASCs group (500 μL PBS containing 25 μg aged apoEVs-ASCs per well), and the young apoEVs-ASCs group (500 μL PBS containing 25 μg young apoEVs-ASCs per well). After culturing for 6 h and 24 h, images were captured again by an inverted microscope (Olympus, Japan). The migrated area was measured using ImageJ software (n=3). The percentage of migrated area = [(A_0_ - A_t_)/A_0_] × 100%, where A_0_ and A_t_ are the initial area (t = 0 h) and the remaining migrated area at different time points (t= 6 h and 24 h).

### 2.7 Tube formation assay

To investigate the angiogenesis of endothelial cells induced by aged and young apoEVs-ASCs, endothelial cells were divided into the PBS group, aged apoEVs-ASCs group, and young apoEVs-ASCs group. In the PBS group, cells were cultured in the normal culture medium with PBS. In the aged and young apoEVs-ASCs groups, cells were pretreated with 25 μg/mL aged and young apoEVs-ASCs overnight, respectively. Then, cells were collected, and seeded at 3×10^3^ cells per well into 96-well plates, which were coated with 50 μL Matrigel (Corning, USA). After culturing for 6 h, images were captured by an inverted microscope (Olympus, Japan). The points of nodes and junctions were measured using Image-Pro Plus 6 (n=3).

### 2.8 *In vivo* animal experiments

All operations were performed on 8-week-old male SD rats (n=45) under general anesthesia with 1% pentobarbital sodium (10 mL/kg, intraperitoneal injection). The dorsal area was shaved and sterilized with 75% ethanol. One circular full-thickness skin wound (1.5 cm in diameter) was prepared by resecting according to the circle marked with a pen. The wounds were divided into three groups: PBS group, aged apoEVs-ASCs group, and young apoEVs-ASCs group. In the PBS group, 200 μL PBS was subcutaneously injected around the wounds at once. In the aged and young apoEVs-ASCs groups, 200 μL PBS containing 5 μg aged and young apoEVs-ASCs also were subcutaneously injected around the wounds at once, respectively. Digital photographs were taken on days 0, 4, 7, and 14. The wound area was measured using the ImageJ software. The wound healing rate was calculated according to the formula: D_n_ wound healing rate = (D_0_ wound area - D_n_ wound area)/D_0_ wound area x 100%. Rats were euthanized on day 14, and the samples were harvested for further testing.

### 2.9 Histological staining

For histological staining, mandibular condyles were fixed by freshly prepared 4% paraformaldehyde in PBS (pH=7.4) overnight, decalcified in 14% EthyleneDiamine Tetraacetic Acid (EDTA) at 4℃, and embedded in paraffin. The samples were cut at 4.5 µm thickness and stained with H&E (Solarbio, China), Masson's trichrome (Servicebio, Wuhan, China), and Sirius red (Solarbio, Beijing, China) as previously reported 38.

### 2.10 Immunohistochemical staining

Immunohistochemical staining was performed as previously reported. The concentration of primary antibodies for the immunohistochemistry is alpha-smooth muscle actin (α-SMA, 1:200, Abcam, ab5694), and Immunoreactivity detection was done by a 3, 3-diaminobenzidine (DAB) kit (Solarbio, Beijing, China).

### 2.11 Statistical analysis

All statistical analyses were performed using GraphPad Prism 6 software. All data were reported as mean±SEM. One-way variance (ANOVA) and two-way ANOVA for multiple comparisons were used to determine the level of significance. *p < 0.05, **p < 0.01, ***p < 0.001.

## 3. Results

### 3.1 Aged and young apoEVs-ASCs have similar characteristics

**Figure 1:**
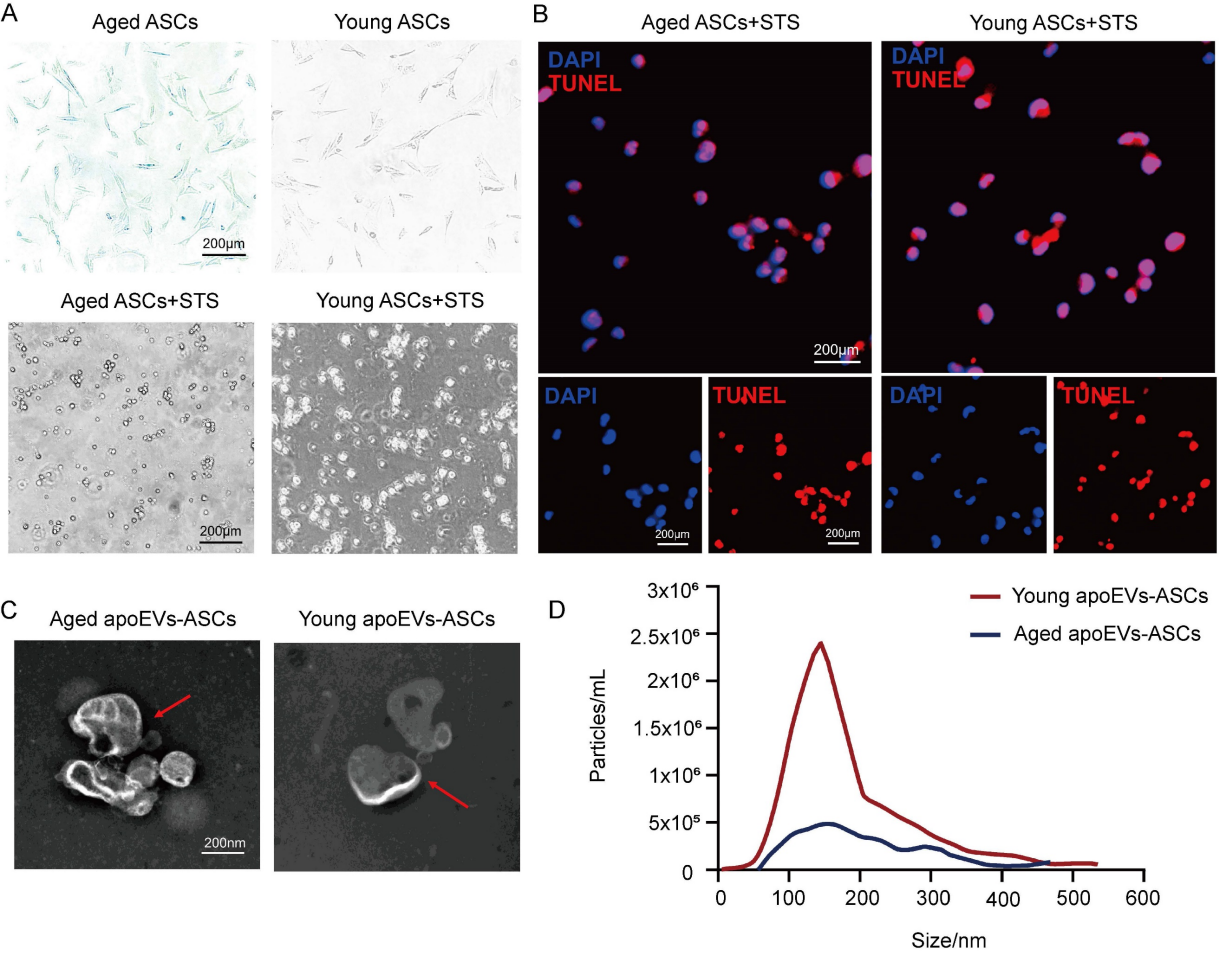
ApoEVs-ASCs from young and aged rats showed similar characteristics. (A-B) Senescence-associated beta-galactosidase staining of ASCs from aged and young groups. Staurosporine-induced apoptosis of ASCs was by morphological alterations and TUNEL assay (Scale bars = 200 nm). (C) Morphology of young and aged apoEVs-ASCs shown by transmission electron microscopy (Scale bars = 200 nm). (D) Size distribution and particle concentration of aged and young apoEVs-ASCs were revealed by nanoparticle tracking analysis.

ASCs were successfully isolated from abdominal subcutaneous adipose tissues from the young (3-week-old) and aged (22-month-old) male rat groups. Staurosporine (STS) was used to induce apoptosis of rat ASCs as previously described. Then apoEVs were isolated using gradient centrifugation as described by Qu et al 37. ASCs derived from both young and aged donors exhibited a typical fibroblast-like morphology. After induction of STS for 16-18 h Senescence-associated beta-galactosidase (SA-β-gal) staining demonstrated β-gal positive cell counts in the young donor group were lower than the count in the aged group (upper panel, Figure 1A). The apoptosis of ASCs from the aged and young donors was confirmed by characteristic morphology changes and TUNEL staining. (Lower panel, Figure 1A, and Figure 1B). Transmission electron microscopy (TEM) analysis showed a typical double-membrane spherical structure in both groups (Figure 1C). By nanoparticle tracking analysis (NTA), aged and young apoEVs-ASCs have similar size distribution profiles, which were both concentrated between 50 and 400 nm (average size 156.2 nm and 148.7 nm, respectively) (Figure 1D). These results suggested that aged and young apoEVs-ASCs had similar profiles in physical and phenotypic characteristics.

### 3.2 Young apoEVs-ASCs displayed significant beneficial effects in full-thickness skin wound healing

**Figure 2:**
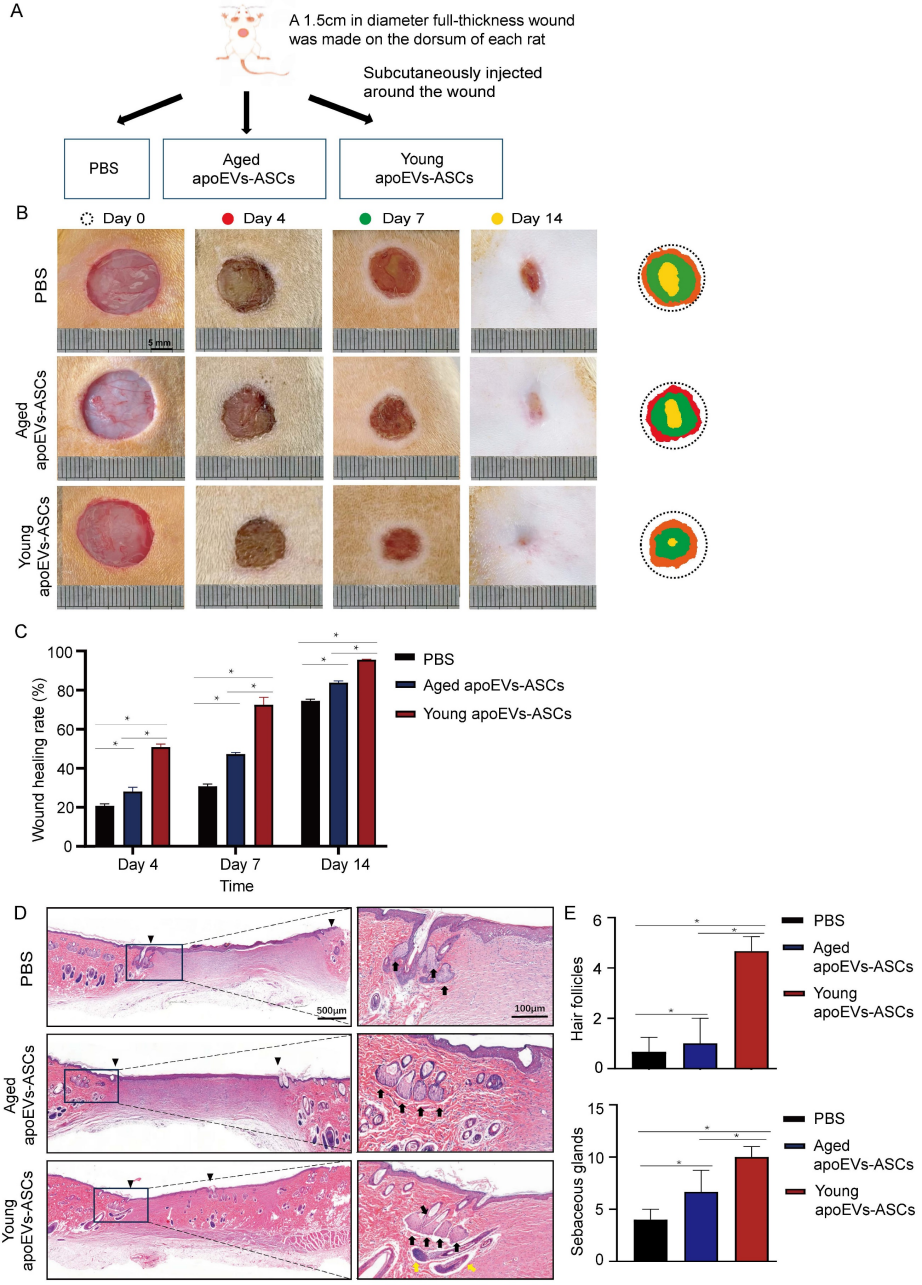
Young apoEVs-ASCs exhibit superior ability to accelerate full-thickness skin wound healing. (A) Schematic view of the experimental operation process. (B) Representative digital photographs of wound areas on day 0, day 4, day 7, and day 14. Pattern graphs of wound areas were established for each time point. (C) The wound healing rate was analyzed (n=9) (*p<0.05). (D) Representative images of wounds on day 14 with H&E staining. The black inverted triangles pointed out the edge of scars and the black dotted boxes pointed out the area of regenerated hair follicles and sebaceous glands (Scale bar = 500 μm). In the right magnified panels, yellow and black arrows mark the presence of hair follicles and sebaceous glands (Scale bar = 100 μm). (E) Quantitative analysis of hair follicles and sebaceous glands (n=3) (*p<0.05).

To determine the effects of aged and young apoEVs-ASCs on skin wound healing *in vivo*, one full-thickness skin wound (1.5cm in diameter) was established in a rat model. In the PBS group, 200 μL PBS was subcutaneously injected around the wound once as a control to support comparison. In the aged and young apoEVs-ASCs groups, 200 μL PBS containing 5 μg aged and young apoEVs-ASCs respectively also were subcutaneously injected around the wounds once (Figure 2A). The wounds were photographed at four time points (day 0, day 4, day 7, and day 14) to discover the wound healing rate. Pattern graphs were established to indicate the edge of wounds more clearly. The dotted circle represented the edge of initial wounds at day 0 and the colored parts represented the area of unhealed wounds at different time points (Figure 2B). Wound healing rate (%) was defined as initial wound area-unhealed wound area at day X / initial wound area at day 0 ×100%. After statistics, these findings suggest that compared to aged apoEVs-ASCs and PBS groups, young apoEVs-ASCs exhibit the strongest ability to accelerate skin wound healing. Based on the results on day 4 and 7, wound healing rate was almost doubled in young apoEVs-ASCs group (52.26% ± 1.57%, 74.56% ± 3.94%) compared to the aged apoEVs-ASCs (28.86% ± 2.19%, 48.53% ± 0.83%) and PBS injection group (21.35% ± 0.96%, 31.54% ± 1.21%). On day 14, all full-thickness skin wounds almost healed in the young apoEVs-ASCs group (98.30% ± 0.22%), in contrast, the wound healing rate in the aged apoEVs-ASCs and PBS groups were 86.26% ± 0.86% and 76.63% ± 0.90% (Figure 2C). In histological analysis, treatment of rats with young apoEVs-ASCs significantly promoted regeneration of hair follicles and sebaceous glands on day 14 after injury compared with the aged apoEVs-ASCs and PBS groups (Figures 2D, E). These results demonstrated the remarkable skin tissue regenerative potential inherent in young apoEVs-ASCs.

### 3.3 Effects of aged and young apoEVs-ASCs on scar formation

**Figure 3:**
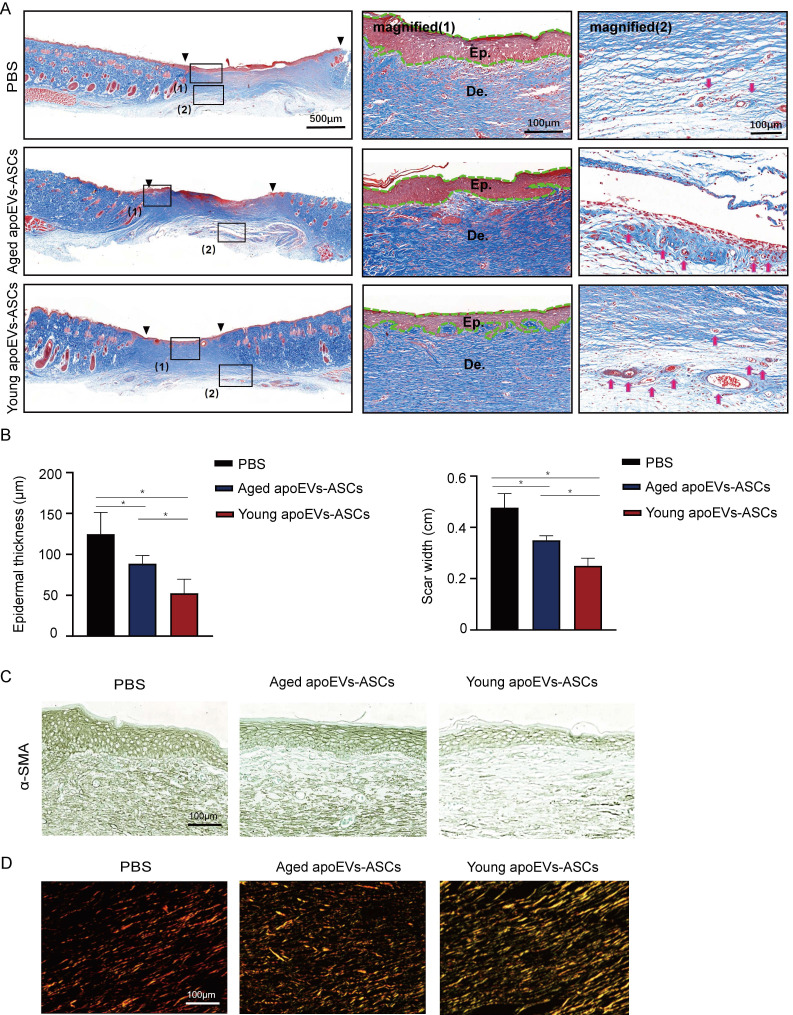
Effects of aged and young apoEVs-ASCs on scar formation. (A) Representative images of scars at day 14 with Masson's staining (Scale bar = 500 μm). The black inverted triangles pointed out the edge of the scar area. The black dotted boxes (1) and (2) were magnified in the middle and right panels. The green lines in the middle panel pointed out the edge of the regenerated epidermis. (Ep.: epidermis; De.: dermis, Scale bar = 100 μm). Red arrows in the right panel indicate the newly formed blood vessels (Scale bar = 100 μm). (B) The epidermal thickness and scar width were analyzed (n=3) (*p<0.05). (C) Representative images of α-SMA immunohistochemical staining (Scale bar = 100 μm). (D) The polarized microscopy images of Sirius red staining showed a decreased ratio of collagen I/ collagen III in the aged and young apoEVs-ASCs groups (middle and right panels, Scale bar = 100 μm).

In addition to the wound healing rate and skin appendage regeneration, scar formation is also a key parameter to evaluate the quality of wound healing. As shown in Masson's staining, the narrowest scar widths and thinnest epithelial were observed in the young apoEVs-ASCs group at day 14 after injury, compared with the aged apoEVs-ASCs and PBS group. We also found that scar width and epithelial thickness were significantly reduced in the aged apoEVs-ASCs group, compared to the PBS group, demonstrating the anti-scar formation ability of aged apoEVs-ASCs (Figures 3A, B). Furthermore, increased newly formed vessels were also observed in the young apoEVs-ASCs treated wound (Figure 3A, right panel).

Myofibroblasts play a pivotal role in the repair of damaged skin tissue and are engaged in forming scar tissue by producing and organizing collagen/extracellular matrix (ECM). Alpha-smooth muscle actin (α-SMA) serves as a key marker of myofibroblasts, particularly during wound healing processes. So, we further performed α-SMA immunohistochemical staining. The result showed the expression of α-SMA gradually reduced in the PBS, aged, and young apoEVs-ASCs groups, with the least expression in the young apoEVs-ASCs group (Figure 3C).

Fibers constitute the primary component of scars, and the analysis of their composition involves Sirius red staining. The polarized light images showed reduced collagen I (orange-red birefringence) and increased collagen III (green-colored birefringence) in the aged and young apoEVs-ASCs groups (Figure 3D). This shift in collagen types was more pronounced in the young apoEVs-ASCs group, which was associated with a notable reduction in scar formation and an enhancement in skin softness.

These results suggested that both aged and young apoEVs-ASCs have the potential to reduce scar formation, which was attributed to several factors, including increased angiogenesis, regulation of myofibroblast activation, and alterations in collagen composition. Notably, young apoEVs-ASCs showed superior scar-reducing abilities compared to their aged counterparts.

### 3.4 Young apoEVs-ASCs promoted proliferation and migration of fibroblasts

To understand the mechanism of the differential effects on promoting skin wound healing between aged and young apoEVs-ASCs, *in vitro* experiments were conducted using primary fibroblasts and endothelial cells.

**Figure 4:**
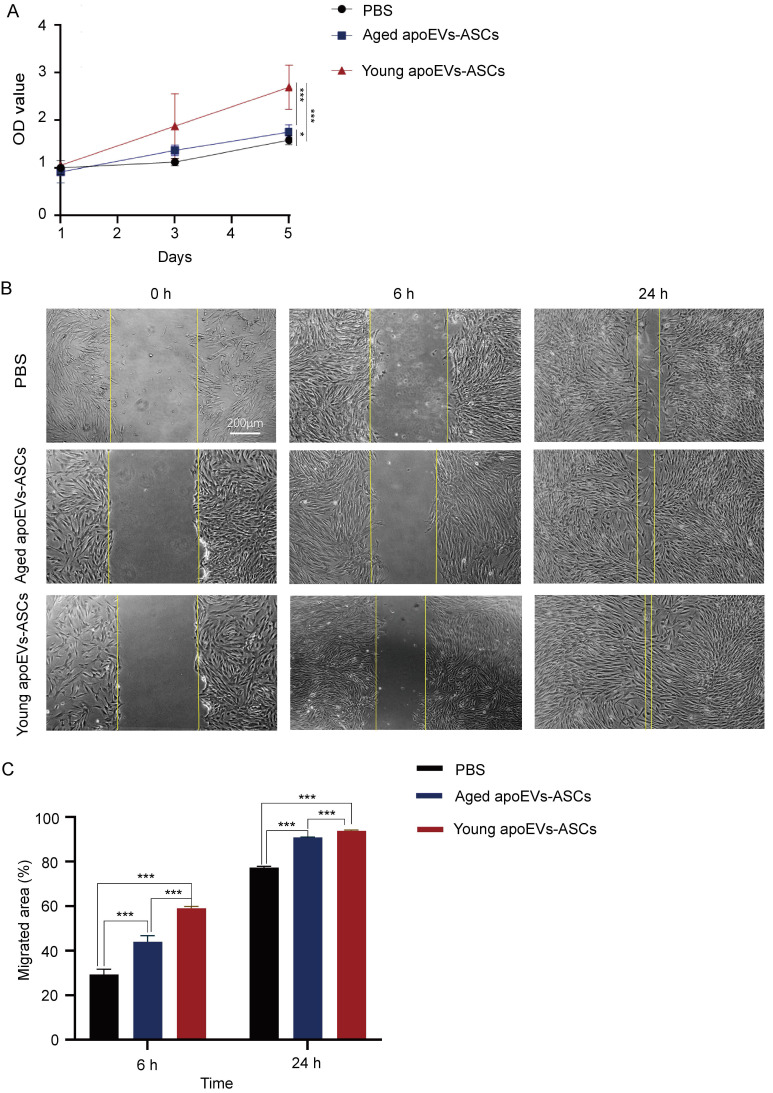
The effects of aged and young apoEVs-ASCs on the proliferation and migration of fibroblasts. (A) The proliferation of fibroblasts was measured by the CCK-8 assay (n=5) (*p<0.05, ***p<0.001). (B) Representative images of migrated area at 6 h and 24 h in scratch assay. The yellow lines pointed out the edge of migrated cells (Scale bar = 200 µm). (C) Migrated area (%) of fibroblasts at 6 h and 24 h were analyzed (n=3). (***p<0.001).

Growth curves of fibroblasts co-cultured with young and aged apoEVs-ASCs at 25μg/ mL were plotted with CCK-8 assay (Figure 4A). The results of the CCK-8 assay showed that fibroblast growth ability increased in the aged and young apoEVs-ASCs group, compared with the PBS group. The fibroblast proliferation decreased with the increase in age. The difference was statistically significant on day 5.

In the cell scratch assay, the migration abilities of fibroblasts in the PBS group, aged, and young apoEVs-ASCs groups were detected. Compared to the PBS group, the migration of fibroblasts into scratched areas in the young apoEVs-ASCs group increased by 2-fold and 1.21-fold after 6 and 24 h, respectively. And 1.5-fold and 1.17-fold increases in fibroblast migration rates were observed in the aged apoEVs-ASCs group after 6 and 24h, respectively, relative to the PBS group (Figures 4B, C). Collectively, these results indicate that young apoEVs-ASCs can significantly promote the proliferation and migration of fibroblasts *in vitro*.

### 3.5 ApoEVs-ASCs from young and aged rats promoted endothelial cell proliferation, migration, and angiogenesis *in vitro*

**Figure 5:**
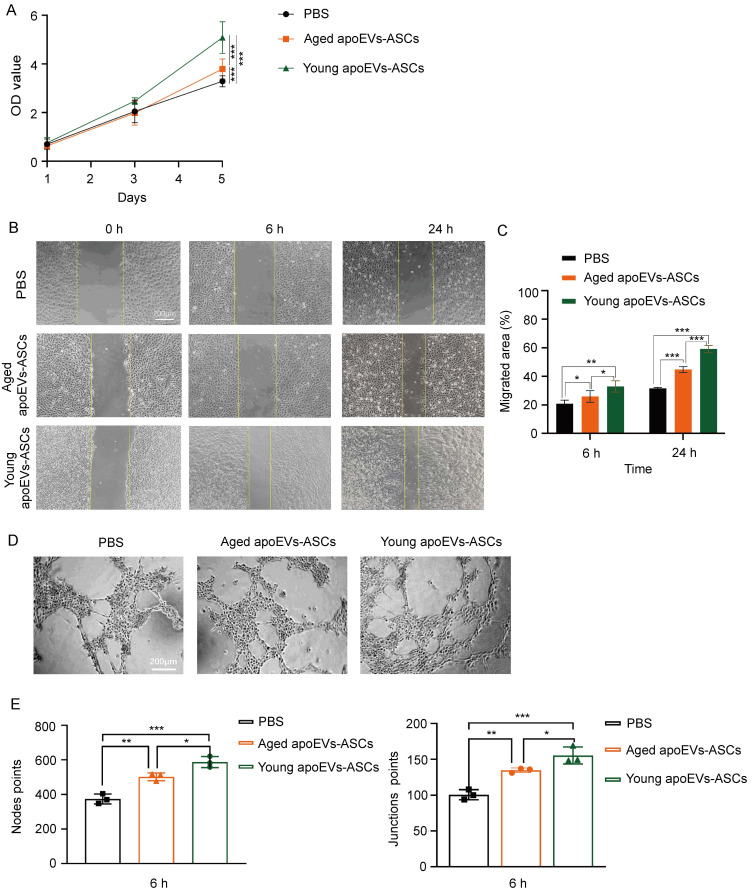
The effects of aged and young apoEVs-ASCs on the proliferation, migration, and angiogenesis of endothelial cells. (A) The proliferation of endothelial cells was measured by the CCK-8 assay (n=5) (***p<0.001). (B) Representative images of migrated area at 6 h and 24 h in scratch assay. The yellow lines pointed out the edge of migrated cells. (Scale bar = 200 µm.) (C) Migrated area (%) of endothelial cells at 6 h and 24 h were analyzed (n=3) (*p<0.05, **p<0.01, ***p<0.001). (D) Representative images of tube-like structure formation of endothelial cells. (Scale bar = 200 µm.) (E) The node points and junction points of view (scale bar = 200 µm) were analyzed (n=3) (*p<0.05, **p<0.01, ***p<0.001).

Similar to the behaviors of fibroblasts, both CCK-8 assay and scratch assay results demonstrated that 25μg/mL young and aged apoEVs-ASCs could significantly enhance the proliferation (on day 5) and migration (on 6 h and 24 h) of endothelial cells, compared to the PBS groups (Figures 5A, B, C). Furthermore, tube formation assay showed that young and aged apoEVs-ASCs could significantly induce the angiogenesis of endothelial cells, based on the increasing number of the points of nodes and junctions (Figures 5D, E). In contrast to young apoEVs-ASCs, the effects of aged apoEVs-ASCs on promoting endothelial cell proliferation, migration, and angiogenesis *in vitro* were less pronounced. Taken together, young apoEVs-ASCs contributed to high-quality skin wound healing, which might be related to the positive effects on regulating the behavior of endothelial cells *in vitro*.

## 4. Discussion

The present study demonstrates that young apoEVs-ASCs achieved more effective therapeutic outcomes in a rat model of skin injury. Aged apoEVs-ASCs are deficient in promoting skin wound healing and decreasing scar formation. Mechanistically, young apoEVs-ASCs significantly induce proliferation, migration, and angiogenesis of endothelial cells and promote proliferation and migration of fibroblasts compared with their aged counterparts.

The skin is the most vulnerable tissue in the human body. Therefore, therapies for tissue regeneration receive increasing attention. ASCs isolated from the abdominal subcutaneous fat have gained vast attention due to the ease of tissue harvesting procedure, highly abundant fat tissue, fast healing of the donor site, and ease of isolation 39. Accumulating evidence supports that apoEVs derived from stem cells show great potential in skin wound repair via paracrine pathways. However, donor age may affect the quantity and quality of mesenchymal stem cells, which may influence their tissue repair ability. Nevertheless, the effect of the donor's age on the biological characteristics and cellular function of ASCs is controversial. Park *et al.* discovered that age may affect the cellular function of ASCs. Qu *et al.* 40 demonstrated that age affects the proliferation and migration but not the adipogenic differentiation potential of ASCs. On the contrary, Horinouchi *et al.* 41 found that age does not seem to significantly affect the cell division or adipogenic or osteogenic differentiation ability of ASCs isolated from lipoaspirates. Dufrane D 42 reported that cellular senescence, the yield of ASCs isolation, and the secretome of ASCs were unaffected by age. In addition, Ding *et al.* 43 revealed promising proliferation and differentiation capabilities of ASCs regardless of the donor's age. Thus, it is unknown whether donor age affects the regenerative potential of apoEVs-ASCs in an animal model of cutaneous skin injury. In this study, we first tried to isolate apoEVs-ASCs from young and aging donors to validate their efficacy in wound repair using a rat model.

This study evaluated the therapeutic effect of young and aging apoEVs-ASCs on full-thickness skin wounds with a diameter of 1.5 cm. The present study revealed that aged apoEVs-ASCs do not possess similar skin regenerative capacity as the young counterparts in a rat model of skin injury. Several studies have demonstrated that angiogenesis of endothelial cells plays a significant role in inducing high-quality skin wound healing 44-46. Compared with other stem cells, abdominal ASCs secrete more growth factors, such as vascular endothelial growth factor (VEGF), hepatocyte growth factor (HGF), basic fibroblast growth factor (FGF-2), epidermal growth factor (EGF), which facilitate angiogenesis and re-epithelization 47. In a dental pulp extraction model, Li *et al.* found that apoEVs derived from human deciduous pulp stem cells activated endogenous endothelial cells autophagy and caused angiogenesis by transferring mitochondrial translation elongation factor Tu 48. Dong et al also confirmed the angiogenic effect of apoEVs derived from adipose tissue 32. Our results showed that young apoEVs-ASCs significantly promote proliferation, migration, and angiogenesis of endothelial cells *in vitro* compared with aged apoEVs-ASCs. This finding might explain the observed difference in skin appendage regeneration between aged and young apoEVs-ASCs.

As a major component of the dermis, fibroblasts play an essential role in the skin wound repair and collagen remodeling process 49. In this study, we revealed that aged apoEVs-ASCs promoted the proliferation and migration of fibroblasts *in vitro* but had a lower level compared with young apoEVs-ASCs. Previous studies have demonstrated that ASCs-derived exosomes exert their anti-scar formation effects by regulating fibroblasts and myofibroblasts 50. Collagen remodeling is a dynamic process, and the composition of collagen in wound healing plays a crucial role. Studies have shown that a lower ratio of thick collagen I and relatively small collagen indicates less scar formation 51. Our results showed that scar formation was significantly reduced in the young apoEVs-ASCs group, which is related to the decreased ratio of collagen I/III and expression of α-SMA. In the present study, we found that the young apoEVs-ASCs group exhibits a stronger influence on the shift in collagen types during tissue repair, emphasizing its potential to promote high-quality wound healing and scar reduction.

Despite these results comparing the therapeutical potential of aged and young apoEVs-ASCs, there are some limitations. Further studies are required to explore the mechanisms through which young apoEVs-ASCs promote skin wound healing.

## 5. Conclusions

In conclusion, we first compared *in vitro* characteristics and the therapeutic effects of the administrated aged and young apoEVs-ASCs in skin wound repair. Young apoEVs-ASCs showed a superior ability to accelerate the full-thickness skin wound healing process and reduce scar formation, which could be attributed to a significantly higher proliferation, migration of fibroblasts and endothelial cells, and increased neo-angiogenesis activity. Our data suggest that apoEVs-ASCs from young donors should be employed for the treatment of cutaneous wounds.
